# Physical Cultivation

**Published:** 1854-04

**Authors:** 


### PHYSICAL CULTIVATION.

All must admit that the bodily habits and occupations of the young have a material influence on their physical development. Contrast the printer with the blacksmith, the tailor with the hunter, the working farmer with the student. If near relations marry each other for a very few generations, the invariable result is bodily deformity and mental imbecility, and if persevered in, the very race and name die out. This is one of the important causes of the decline and fall of nations; it is a law of nature whose infraction is visited with punishment—signal—infallible. The practical remedy is opposite marriages, the city should marry the country, the south the north; the sea-shore should marry the interior, the plain should wed the mountain-top; districts should marry wide asunder. This may be the reason that the Patriarchs were sent far from home to marry. It is very certain that the cultivation of the physical man with a view to its more perfect developement, on rational principles, would elevate the race bodily, mentally, morally. A vigorous body rightly educated gives a vigorous intellect, and give this intellect bible teaching, and it becomes the highest type of a Christian—the Christian from principle, the only man in the wide universe who can be depended on. The following article is a step forward :—

" Baby Exhibition.—The Committee of the Southern Central Agricultural Association have been authorized to offer the fol lowing premiums, to be awarded at the next fair in Augusta, Ga., January, 1854.

" 1st Premium : Silver Pitcher, $50 for the handsomest and finest babe two years old.

" 2d Premium : Silver Pitcher, $25 for the handsomest and finest babe one year old.

" 3d Premium : Silver Goblet, $10 for the handsomest and finest babe six months old.

" The children to be clothed in domestic fabrics ; the premiums awarded under the direction of the Executive Committee."

